# The Artificial-Liver Blood-Purification System Can Effectively Improve Hypercytokinemia for COVID-19

**DOI:** 10.3389/fimmu.2020.586073

**Published:** 2020-12-23

**Authors:** Jing Guo, He Xia, Shuting Wang, Liang Yu, Huafen Zhang, Jun Chen, Ding Shi, Yanfei Chen, Yan Zhang, Kaijin Xu, Xiaowei Xu, Jifang Sheng, Yunqing Qiu, Lanjuan Li

**Affiliations:** State Key Laboratory for Diagnosis and Treatment of Infectious Diseases, First Affiliated Hospital, College of Medicine, Zhejiang University, Hangzhou, China

**Keywords:** artificial-liver blood-purification system, hypercytokinemia, COVID-19, ARDS, IP-10

## Abstract

Since the December 2019 outbreak of coronavirus disease 2019 (COVID-19) in Wuhan, the infection has spread locally and globally resulting in a pandemic. As the numbers of confirmed diagnoses and deaths continue to rise, COVID-19 has become the focus of international public health. COVID-19 is highly contagious, and there is no effective treatment yet. New treatment strategies are urgently needed to improve the treatment success rate of severe and critically ill patients. Increasing evidence has shown that a cytokine storm plays an important role in the progression of COVID-19. The artificial-liver blood-purification system (ALS) is expected to improve the outcome of the cytokine storm. In the present study, the levels of cytokines were detected in 12 COVID-19 patients pre- and post-ALS with promising results. The present study shows promising evidence that ALS can block the cytokine storm, rapidly remove the inflammatory mediators, and hopefully, suppress the progression of the disease, thereby providing a new strategy for the clinical treatment of COVID-19.

## Introduction

The coronavirus disease 2019 (COVID-19) is caused by the new severe acute respiratory syndrome coronavirus 2 (SARS-CoV-2). The outbreak of COVID-19 was reported in Wuhan, China, in December 2019. Since then, the infection spread rapidly worldwide. According to the World Health Organization (WHO) report, there have been 25,118,689 confirmed cases of COVID-19, including 844,312 deaths, up to August 31, 2020. At present, the overall mortality of COVID-19 is about 1.36%–15% (1, 2). According to recent reports, humans can be infected by the SARS-CoV-2 for a second time. So far, there are no effective treatments available for COVID-19, and the development of a COVID-19 vaccine is underway.

Mounting evidence suggests that a hyper-inflammatory immune response in critically ill patients is responsible for acute respiratory distress syndrome (ARDS) and multiorgan failure (3, 4). A cytokine storm, also called hypercytokinemia, is also an important cause of death in SARS, MERS, H5N1, and H7N9 infections (3–5). Hence, the timely control of cytokine storms and reduction in inflammatory cell infiltration in the lungs are key to reducing COVID-19-related deaths (6). Multiple therapeutic strategies, such as antibody therapies with tocilizumab, sarilumab, and siltuximab and blood-purification techniques (BPT), including therapeutic plasma exchange (TPE), absorption, perfusion, and blood plasma filtration, among others, might potentially be effective for COVID-19 treatment (7–10).

The present agents used to modulate the immune response mainly include glucocorticoids, intravenous immunoglobulin, and cytokine antagonists (6). However, a large number of clinical studies have shown that corticosteroids exhibit no efficacy in reducing mortality in the treatment of SARS and MERS. On the contrary, these delayed virus clearance and resulted in various complications (11, 12). Furthermore, the intravenous injection of immunoglobulin or IL-6R-related antibodies also requires further evaluation (13). Systemic corticosteroid administration has also been proven ineffective for the treatment of COVID-19. It has been clearly recognized that immunosuppression may be a double-edged sword in the treatment of COVID-19 (14). Thus far, only a limited number of studies have been reported on the use of BPT in the treatment of COVID-19 patients; although the effectiveness of BPT has been demonstrated in a few studies, it remains controversial (10, 15–20). TPE was performed in patients with severe COVID-19 infection in three small studies (9, 21, 22). After treatment, sequential organ failure assessment (SOFA) score; oxygenation index; lymphocyte count; and serum levels of total bilirubin, lactate dehydrogenase, ferritin, C-reactive protein, and interleukin-6 (IL-6) were all decreased, and no adverse reactions were detected. Due to limited study funding, evaluation of cytokine clearance was mostly focused on IL-6 in most studies. These studies reported higher fatality rates, and some reported no improvement in patient mortality rates (23, 24).

The artificial-liver blood-purification system (ALS) consists of modules for plasma replacement, plasma adsorption, and blood/plasma filtration, and can effectively remove cytokines from the blood. This mainly has been used for the treatment of liver failure and has significantly reduced the mortality of these patients (25). Investigators have also successfully used ALS to remove cytokines in the treatment of critically ill H7N9-infected patients (26). Based on the similar pathological mechanism, it is expected that ALS may also be useful in the treatment of severe and critically ill COVID-19 patients. Hence, we aimed to verify the therapeutic effect of ALS in 12 COVID-19 patients in this study.

## Methods

The present study included 12 critically ill COVID-19 patients, who received treatment with ALS from January 15, 2020, to March 31, 2020. Each patient’s sex, age, symptoms, complications, and disease severity were recorded in detail. Written informed consent was obtained from each patient or a legally authorized representative. The present study was approved by the institutional review board of the First Affiliated Hospital, School of Medicine, Zhejiang University.

Each patient underwent three consecutive courses of ALS, and peripheral blood was collected before and after each course (pre-1st ALS, post-1st ALS, pre-2nd ALS, post-2nd ALS, pre-3rd ALS, and post-3rd ALS). The mode we adopted here is mainly plasma exchange and hemoperfusion. Each hemoperfusion lasted for 3 h, and the amount of plasma exchange volume was about 2000 ± 50 mL. The details are shown in **Table 1**. The venous blood was centrifuged at 3000 rpm for 5 min and then stored at -80°C until assayed.

**Table 1:** ALS therapy mode for 3 consecutive courses.

Group	Therapy mode of each ALS	Number	Duration of each course	Interval between each course
	1st ALS/2nd ALS/3rd ALS		1st ALS/2nd ALS/3rd ALS	1st ALS-2nd ALS/2nd ALS-3rd ALS
HP group	HP/HP/HP	6	3 h/3 h/3 h	21 ± 0.5h/21 ± 0.5h
PE+HP group	PE+HP/PE+HP/PE+HP	4	3.5 ± 0.2h/3.5 ± 0.2h/3.5 ± 0.2h	20.5 ± 0.5h/20.5 ± 0.5h
PE group	PE/PE/PE	1	1.5 ± 0.2h/1.5 ± 0.2h/1.5 ± 0.2h	22.5 ± 0.5h/22.5 ± 0.5h
PE+HP/HP/HP	PE+HP/HP/HP	1	3.5 ± 0.2h/3 h/3 h	20.5 ± 0.5h/21 ± 0.5h

PE, plasma exchange; HP, hemoperfusion.

The magnetic bead–based multiplex immunoassays were developed using the Bio-Plex Pro™ Human 48-plex Cytokine Screening Panel, according to the manufacturer’s instructions, using the Bio-Plex 200 suspension array system (Bio-Rad, Hercules, CA, USA) in a BSL-2 laboratory. The primary data were analyzed using the Bio-Plex Manager Software, version 6.1.1 (Bio-Rad, Hercules, CA, USA).

### Statistical Analysis

All statistical analyses were performed using GraphPad Prism 5 software (GraphPad Software, San Diego, CA, USA), SPSS20.0 (IBM, Armonk, NY, USA), and R-3.6.3 (available from: http://www.r-project.org/). The results of the cytokines were expressed as medians. The paired serum cytokine results generated at different time points for the same patient were analyzed using the Wilcoxon signed rank test. Other continuous variables were expressed as the mean and standard deviation (SD) or were transformed as grade variables for clinical scale estimation. The data between groups were compared by two-sample *t*-test or two-paired sample *t*-test. Grade variables were expressed as the mean rank. For nonparametric data, the Friedman and Nemenyi rank sum tests (From R package PMCMR, Pohlert T, 2014) were used for analyses of Acute Physiology and Chronic Health Evaluation (APACHE) II, pneumonia severity index (PSI), and SOFA scores. P<0.05 was considered to indicate statistical significance.

## Results

### Patient Information

Clinical data on the 12 patients are presented in **Table 2**. There were 10 men and 2 women with a mean age of 62 years (range: 36–90 years). The patients’ coexisting diseases included hypertension (9/12), diabetes (3/12), coronary heart disease (1/12), and arrhythmia (2/12). The incidence of ARDS and renal dysfunction was 12/12 and 4/12, respectively. All 12 patients were admitted to the ICU; of these, seven received mechanical ventilation, and three received extracorporeal membrane oxygenation (ECMO) treatment.

**Table 2:** Characteristics of patients treated by ALS.

Characteristics*	Patients (n=12)
**Age** years	62 ± 14.27
**Male** n%	10(87%)
**BMI** (kg/m^2^)	23.90 ± 2.62
**Selected presenting signs and symptoms**	
**Fever**	10(87%)
**Cough**	8(66%)
**Diarrhea**	4(33%)
**Shortness of breath**	9(75%)
**Myalgia**	5(42%)
**Headache**	3(25%)
**Bacterial/Fungi infection**	3(25%)
**Comorbidites**	
**Coronary heart disease**	1(8%)
**Chronic liver disease**	1(8%)
**Arrhythmia**	2(17%)
**Hypertension**	9(75%)
**Renal dysfunction**	4(33%)
**Diabetes mellitus**	3(25%)
**Requiring MV**	7(58%)
**Requiring ECMO**	3(25%)
**Drug treated**	
**Ambroxol**	10(87%)
**Vasopressor**	5(42%)
**(Adr, NE, Dopamine, Dobutamine)**	
**Antibiotic**	11(92%)
**Immunogloblin**	10(87%)
**Glucocorticoids**	12(100%)

*Data was collected at the first admission hospital.

### Cytokine Changes in Serum

**Figure 1:**
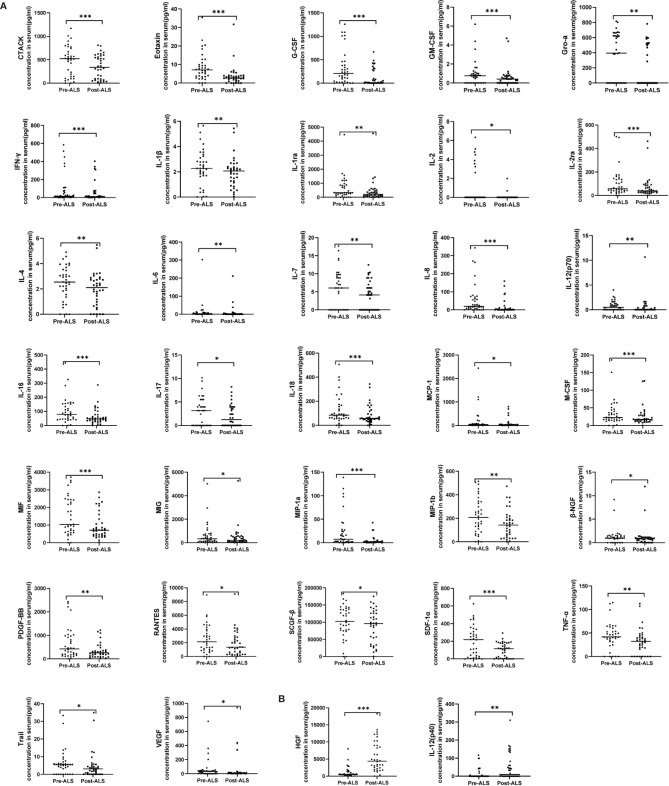
Serum cytokine levels in COVID-19 patients pre- and post-ALS analyzed through a paired study. Pre-ALS represents the cytokine levels before the three courses of ALS in the 12 critically ill COVID-19 patients. Post-ALS represents cytokine levels after three courses of ALS in the 12 patients. The changes of cytokine levels pre- and post-ALS were analyzed by a paired study. In all, 34 of 48 cytokines exhibited significant changes post-ALS. **(A)** The levels of 32 cytokines decreased post-ALS by paired studies. **(B)** The levels of two cytokines increased post-ALS by paired studies. The middle line in the figure is presented as median values. *P < 0.05, **P < 0.01, ***P < 0.001.

Three courses of ALS over three consecutive days were formulated for each patient. Three pairs of blood samples were collected before and after ALS from each patient (pre-ALS: within 1 h before each course of ALS; post-ALS: within 1 h after each course of ALS); 36 pairs of samples were collected in total from all 12 patients. To obtain an overall impression of the therapeutic effect after ALS treatment, we matched data from COVID-19 patients before and after the 36 courses of ALS for a paired analysis. The levels of 34 cytokines exhibited significant changes by paired analysis. Thirty-two cytokines, including IL-6 and TNF-α, were significantly decreased after ALS treatment. The remaining two cytokines—including HGF and IL-12 (p40)—were significantly upregulated (**Figure 1**).

**Figure 2:**
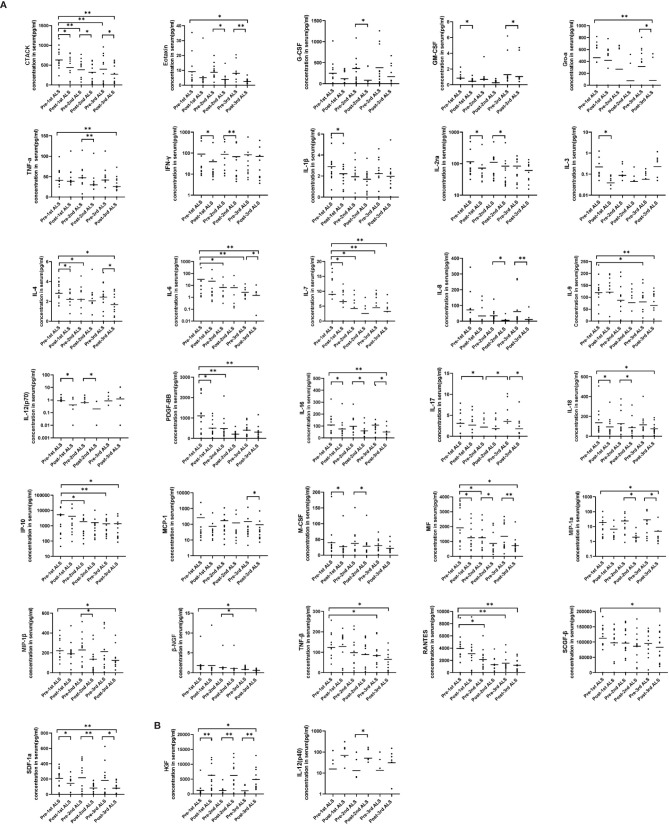
Serum cytokine levels in the COVID-19 patients before and after each course of ALS. Six groups representing the cytokine levels pre-1st, post-1st, pre-2nd, post-2nd, pre-3rd, and post-3rd course of ALS were analyzed. **(A)** The levels of 31 cytokines decreased post-ALS. **(B)** The levels of two cytokines increased post-ALS. The middle line in the figure is presented as median values. *P < 0.05, **P < 0.01.

To further investigate the trend of cytokines in COVID-19 patients with ALS intervention in detail, we show cytokine levels at 6 time points (pre-1st ALS vs. post-1st ALS, pre-2nd ALS vs. post-2nd ALS, pre-3rd ALS vs. post-3rd ALS) in **Figure 2**. The three sets of data before and after 3 courses of ALS were analyzed using paired analysis. Thirty-three of the cytokines exhibited significant changes in response to ALS, which included 31 that were significantly decreased and two [HGF and IL-12 (p40)] that were significantly upregulated. The results also confirmed the therapeutic effect of ALS on removing cytokines. Notably, HGF was significantly upregulated after each treatment.

By pairing analysis, 15 cytokines were found to be significantly decreased after the first course of ALS (pre-1st ALS vs. post-1st ALS, **Figure 2**), including IL-1β and IFN-γ. Among these 15, the levels of five remained low before the second course (post-1st ALS vs. pre-2nd ALS). A total of 16 cytokines significantly decreased after the second course of ALS (pre-2nd ALS vs. post-2nd ALS). In all, the levels of 20 cytokines, including IL-6 and TNF-α, significantly decreased after three courses of treatment (pre-1st ALS vs. post-3rd ALS). These data indicate that ALS treatment significantly alleviated the cytokine storm.

### Clinical Index

Clinical data was recorded at three time points (pre-1st ALS, post-3rd ALS, 1 week post-3rd ALS) for COVID-19 patients. The APACHE II, PSI, and SOFA scores were calculated pre-1st ALS and post-3rd ALS. As shown in **Table 3**, the APACHE II (p=0.0031), PSI, and SOFA scores were significantly decreased after three consecutive days’ treatment with ALS. The APACHE II (p=0.0015), PSI (p=0.0044), and SOFA (p=0.001) scores were also decreased significantly 1 week post-3rd ALS.

**Table 3:** Statistical tests and comparison between groups.

	Mean Rank			Friedman Rank Sum test			p-Value of Nemenyi test		
	Pre-ALS	Post-ALS	1 Week Post-ALS	Chi-square	df	p-Value	Pre-ALS vs. Post-ALS	Pre-ALS vs. 1 Week Post-ALS	Post-ALS vs. 1 Week Post-ALS
APACHII	2.92	1.58	1.5	15.826	2	**3.66E-04**	**0.0031**	**0.0015**	0.9773
PSI	2.67	1.96	1.38	11.476	2	**3.22E-03**	0.1921	**0.0044**	0.326
SOFA	2.79	1.88	1.33	15.268	2	**4.84E-04**	0.064	**0.001**	0.38

Pre-ALS: Data not earlier than 3 days before the first treatment with ALS were selected.

Post-ALS: Data not later than 3 days after the last treatment with ALS were selected.

1 Week Post-ALS: Data 1 week after the last treatment with ALS were selected.

P-values <0.05 are indicated in bold.

As shown in **Table 4**, after three consecutive days’ treatment with ALS, the patients’ PaO_2_ (p=0.0263), PaO_2_/FiO_2_ (p=0.0003) increased, and alveolar-arterial oxygen gradient (A-aDO_2_) (p=0.0014) decreased, and the difference was statistically significant. In addition, the patients’ eGFR (mL/min) (p=0.0350) was elevated. The above results show that ALS treatment can improve both lung and kidney function.

**Table 4:** Clinical characteristics of 12 severe COVID-19 patients pre- and post-ALS.

	Pre-ALS(n=12)*	Sth.(Pre-ALS)	Post-ALS(n=12)**	Sth.(Post-ALS)	p(α<0.05)
**Respiratory Function**					
MAP (mmHg)	95.61	10.07	90.60	14.07	0.0701
PaO_2_ (mmHg)	59.23	10.25	74.76	14.91	**0.0263**
PaCO_2_ (mmHg)	33.44	4.20	37.62	3.78	**0.0037**
FiO_2_	0.56	0.20	0.42	0.12	–
PaO_2_/FiO_2_ (mmHg)	197.27	51.04	229.81	88.32	**0.0003**
A-aDO_2_ (mmHg)	390.65	125.81	189.53	59.64	**0.0014**
Lactin acid (mmol/L)	1.50	0.57	1.98	0.42	0.1362
**Blood Rt**					
WBC (10^9^/L)	9.80	5.15	10.75	3.17	0.2460
RBC (10^12^/L)	4.20	0.60	3.22	0.66	**1.10E-05**
Hemoglobin (g/L)	127.92	18.32	97.33	19.62	**3.16E-05**
Platelet (10^9^/L)	174.00	49.26	115.58	55.21	**0.0088**
Neurtophil (10^9^/L)	8.73	5.01	9.85	3.14	0.2420
Lymphocyte (10^9^/L)	0.68	0.26	0.82	1.08	0.5314
**Coagulation Function**					
D-Dimer (ug/L FEU)	618.08	470.11	2662.42	1654.97	**1.23E-03**
INR	1.02	0.07	1.00	0.11	0.8558
PT (s)	12.28	0.82	12.01	1.27	0.8900
APTT (s)	34.01	5.91	31.44	12.80	0.4265
Fibrinogen (g/L)	5.25	0.76	2.70	1.45	**0.0012**
**Biochemical Examination**					
ALB (g/L)	32.53	4.17	33.11	2.09	0.4357
GLB (g/L)	33.07	10.74	24.40	7.48	**0.0372**
ALT (U/L)	22.08	9.00	37.25	24.72	0.1042
AST (U/L)	31.25	18.94	29.08	14.14	0.7329
GGT (U/L)	114.92	262.10	64.17	65.30	0.4286
ALP (U/L)	102.83	179.63	52.00	25.65	0.3160
LDH (U/L)	323.42	80.95	312.82	55.23	0.5543
hDBH (U/L)	261.25	63.11	259.36	46.92	0.3222
CK (U/L)	137.75	129.72	135.00	106.79	0.7745
CK-MB (U/L)	21.48	10.88	20.91	4.23	0.4174
Cr (umol/L)	110.08	72.69	81.83	30.04	0.1405
TB (umol/L)	16.06	16.91	25.93	33.11	0.0854
DB (umol/L)	9.68	13.49	20.00	33.28	0.1310
eGFR (ml/min)	72.80	28.31	84.69	29.52	**0.0350**

*Pre-ALS: Data not earlier than 3 days before the first treatment with ALS were selected.

**Post-ALS: Data not later than 3 days after the last treatment with ALS were selected.

P-values <0.05 are indicated in bold.

RBC (p=1.10E-05) and hemoglobin levels (p=3.16E-05) dropped significantly after three consecutive days’ treatment with ALS (1012/L). The platelet levels significantly decreased (p=0.0088), and the D-dimer levels (p=1.23E-03) increased.

## Discussion

ARDS is considered to be the leading cause of death in COVID-19 (27). Imbalance between coagulation and inflammation is a predominant characteristic of ARDS, leading to extreme inflammatory response and diffuse fibrin deposition in vascular capillary beds and alveoli (28). Cytokine storms triggered by viral infections can cause endothelial damage/dysfunction and dysregulation of coagulation, which consequently alters microvascular permeability to induce tissue edema and shock, ultimately resulting in acute lung injury (ALI) and ARDS (29, 30). Therefore, controlling the cytokine storm is critical for successful COVID-19 treatment (6). In a previous study, we used ALS to treat H7N9-infected patients and found that ALS can significantly alleviate the cytokine storm. Hence, we attempted to use ALS in the treatment of the cytokine storm in critically ill COVID-19 patients in this study. A total of 32 cytokines were found to be significantly decreased. ALS treatment could significantly alleviate the cytokine storm. The coronavirus infection is primarily attacked by immune cells, including mast cells (MCs). Activation of MCs secretes IL-1 family members (IL-1, IL-33, IL-6) and TNF (31). IL-1, IL-6, and TNF are the major cytokines that cause the cytokine storm, which further aggravates the inflammatory state (31–34). In our study, we found that the levels of TNF-α and IL-6 significantly decreased after three courses of ALS. The IL-1β level was also decreased after the first course of ALS.

IP-10 plays an important role in inflammation by recruiting a variety of cells to the inflammatory site and interacting with the CXCR3 receptor (35). In animal model studies, the monoclonal antibody against CXCL-10/IP-10 can improve ALI induced by the influenza A (H1N1) virus (35). In the present study, it was found that ALS can significantly reduce the level of chemokine IP-10. Macrophage colony-stimulating factor (M-CSF) is a chemokine that can regulate the survival, proliferation, and differentiation of mononuclear macrophage lines, which is derived from activated macrophages and endothelial cells (36). Treatment with ALS can also significantly reduce the levels of M-CSF. Stem cell growth factor beta (SCGF-β) is an endogenous growth factor that can inhibit bone marrow inflammation, enhance hematopoietic recovery after bone marrow suppression, and reverse inflammation (37). The exhaustion of lymphocytes likely promotes the generation of SCGF-β. After ALS treatment, the level of SCGF-β significantly decreased. Hence, it may be necessary to supplement SCGF-β after ALS treatment, which may increase the level of peripheral blood lymphocytes and improve the patient’s condition.

To our knowledge, this is first report to show that hepatocyte growth factor (HGF) can be significantly increased after ALS treatment. HGF is an interstromal-source pleiotropic growth factor that can promote cell mitosis, growth, maturation, and movement and the tissue formation process and induce angiogenesis when combined with hepatocyte growth factor receptor. Furthermore, HGF can improve vascular endothelial permeability and inflammation, protect endothelial cells (38), and improve the ischemia-reperfusion injury in ALI (39, 40). Furthermore, ALS treatment can increase HGF levels and may play a protective role against lung and liver function damage.

In this study, the APACHE II, PSI, and SOFA scores decreased after three consecutive days’ treatment with ALS. This directly indicates that ALS treatment can indeed improve the condition for COVID-19 patients. After 1 week, the above scores continued to decline. Finally, after three consecutive days’ ALS treatment, the patients’ PaO_2_ and PaO_2_/FiO_2_ increased and A-aDO_2_ decreased. In addition, the patients’ estimated glomerular filtration rate (eGFR) also improved. The above results showed that treatment with ALS mainly improved both lung and kidney function.

Although the present study showed that therapeutic strategies with ALS can significantly reduce a patient’s cytokine levels, the present study is a nonrandomized clinical trial. Hence, it is difficult to provide clear evidence to determine whether ALS could reduce the mortality rate. The immune status of patients of different age and sex was different (41, 42). Owing to the limited sample size, further research in the form of large sample, multicenter, randomized controlled trials is required to clarify the above problems. In conclusion, the present study provides preliminary data to support the application of ALS for the treatment of critically ill COVID-19 patients. We recommend early assessment of COVID-19 patients and timely intervention with ALS to improve the prognosis.

## Data Availability Statement

The original contributions presented in the study are included in the article; further inquiries can be directed to the corresponding author.

## Ethics Statement

The studies involving human participants were reviewed and approved by the Institutional Review Board of the First Affiliated Hospital, School of Medicine, Zhejiang University. The patients/participants provided their written informed consent to participate in this study.

## Author Contributions

JG, SW, HX, and LY contributed equally to this article. LL conceived the project and designed the experiments. JG, HX, and SW performed the experiments. LY, HZ, and JC collected clinical samples and specimens. DS, YC, and YZ collected clinical data. KX, XX, YQ, and JS contributed fruitful discussions and helpful ideas. JG analyzed the data and wrote the initial draft with all authors providing critical feedback and edits to subsequent revisions. All authors contributed to the article and approved the submitted version.

## Funding

This work was funded by the Zhejiang Provincial Natural Science Foundation of China (No. LED20H190001 and 2020C03123).

## Conflict of Interest

The authors declare that the research was conducted in the absence of any commercial or financial relationships that could be construed as a potential conflict of interest.
